# Effects of Artificial Texture Insoles and Foot Arches on Improving Arch Collapse in Flat Feet

**DOI:** 10.3390/s20133667

**Published:** 2020-06-30

**Authors:** Yao-Te Wang, Jong-Chen Chen, Ying-Sheng Lin

**Affiliations:** 1 Information Management, National Yunlin University of Science and Technology, Yunlin 64002, Taiwan; d10123001@gemail.yuntech.edu.tw; 2 Plastic Surgery, National Taiwan University Hospital Yunlin Branch, Yunlin 640203, Taiwan; Y01924@ms1.ylh.gov.tw

**Keywords:** textured insole, arch insole, analysis of variance, plantar pressure, flat feet

## Abstract

The arches of the foot play a vital role in cushioning the impact and pressure generated from ground reaction forces due to body weight. Owing to a lack of normal human arch structure, people diagnosed as having flat feet often have discomfort in the soles of their feet. The results may not only cause inappropriate foot pressure distribution on the sole but also further cause foot injuries. This study heavily relies on a homemade foot pressure sensing device equipped with textured insoles of different heights and artificial arches. This was to explore the extent to which the pressure distribution of the foot in people with flat feet could be improved. A further comparison was made of the effects of using the textured insoles with different heights on two different groups of people diagnosed with flat and normal feet respectively. Sixty-five undergraduate and postgraduate volunteers were invited to receive the ink footprint test for measuring their degrees of arch index. Nine of these 65 had 2 flat feet, 3 had a left flat foot, 5 had a right flat foot, and 48 had 2 normal feet. To ensure the same number of subjects in both the control and the experimental groups, 9 of the 48 subjects who had normal feet were randomly selected. In total, 26 subjects (Male: 25, Female: 1; Age: 22 ± 1 years; height: 173.6 ± 2.5 cm; body mass: 68.3 ± 5.4 kg; BMI: 22.6 ± 1.2) were invited to participate in this foot pressure sensing insoles study. The experimental results showed that the use of textured insoles designed with different heights could not effectively improve the plantar pressure distribution and body stability in subjects with flat feet. Conversely, the use of an artificial arch effectively improved the excessive peak in pressure and poor body stability, and alleviated the problem of plantar collapse for patients with flat feet, especially in the inner part of their hallux and forefoot.

## 1. Introduction

The human foot is not only one of the most complicated structures of the human body but also the most sophisticated organ. Improper use of the feet can lead to walking diseases and can even further affect people’s health. The arches of the feet play a decisive role in buffering impact pressure and are responsible for static and dynamic functional stabilization of the body when standing or walking [1,2,3]. People with flat feet lack an arch to cushion the pressure from the ground, due to an arch collapse problem caused by numerous congenital factors or other acquired predisposing factors [4]. Abnormal pressure leads to foot discomfort of flat feet, which, if not treated, will produce pain and disability [5]. Flat feet is regarded as a contributing factor in a wide variety of medical conditions, including lower limb musculoskeletal pathology, such as Achilles tendonitis, plantar fasciitis, hallux valgus [6], and tenderness of the ligamentous laxity [7].

Macwilliams et al. argue that the foot pressure signals, otherwise known as plantar pressure, can directly or indirectly determine the medical cause of a patient’s diseases [8]. Plantar pressure has been commonly used to not only assess patients with foot complaints but also treat their foot-related injury problems [9,10]. There is a significant relationship found between forelimb lesions and maximum plantar pressure in patients with hallux valgus. The risk factors identified for neuropathic foot ulceration have a positive correlation with any increases in plantar pressure [11,12]. The middle foot peak pressure has a positive correlation with any increases in BMI [13]. In response to these problems, some scholars have also suggested that if the foot pressure is evenly distributed throughout the foot area, it can effectively reduce foot injuries to a greater extent [14]. Based on the findings of these studies, uniform distribution of plantar pressure is thought to be as an important issue and should not be neglected.

Flat feet abnormalities are usually resolved using a number of remedies. Foot insoles have been shown to be the successful treatment to reduce the symptoms of flat feet [15,16]. Adding texture to the upper surface of the shoe insoles can alter gait [17,18], thus enhancing standing balance through enhanced plantar tactile stimulation [19]. Charlie et al. further argue that for those patients wearing a textured insole of 3 mm in height, the problem of asymmetric foot pressure and gait can be reduced [20]. Milner further suggests that medial arch support insoles and shoe modifications help to control symptoms in most patients with flat feet [21]. In addition, foot orthoses might benefit the ankle joint in patients with flat feet [22,23].

To date, no systematic reviews have consolidated and critiqued research into textured and arch insoles used to alleviate symptoms of flat feet. In addition, little attention has been paid to the influence of sensory input on pressure distribution on the plantar surface of the foot. The current study may fill this research gap. Previous foot biomechanical studies recorded plantar pressures and center of pressure (COP) of those with flat feet using Kistler force plates or a plantar pressure analysis system [3,15,17,19,20,22,23]. These are expensive devices. To this end, we designed a low-cost homemade foot pressure sensing device equipped with textured insoles of different heights and artificial arches to explore whether the pressure distribution and body stability in patients diagnosed with flat feet could be improved.

## 2. Subjects, Materials and Methods

### 2.1. Subjects

Sixty-five undergraduate and postgraduate volunteers were invited to receive the ink footprint test for measuring their degrees of arch index (AI). These volunteers, who were diagnosed with no diseases of the foot or lower extremity, were invited to participate in the test. Results from the calculation of the AI showed that most participants did not have flat feet; 48 had two normal feet. Relatively few were diagnosed with flat feet, i.e., nine had two flat feet (group I), eight had only one flat foot (group III and IV). To ensure the same number of subjects in both the control and the experimental groups, nine subjects (group II) were randomly selected from the group members diagnosed with two normal feet. Three subjects were diagnosed with a fallen arch only in the left foot were referred to as Group III, and five subjects having a fallen arch only in the right foot were referred to as Group IV. In total, 26 subjects were invited to participate in this foot pressure sensing insoles study. In addition to obtaining the informed consent from the subjects, approval given by the IRB (Case No. 201805068 RINB/National Taiwan University Hospital/Yunlin, Taiwan) was also obtained.

There are many methods considered adequate to measure fallen arches in individuals. The method of the AI proposed by Cavanagh and Rodgers was employed in this study [24]. The AI is normally assessed by the ratio of the zone of the midfoot to the zone of the entire foot, and the zone of the toes is excluded. People’s feet (excluding the toes) can be divided into three equal sections, namely, the forefoot, midfoot, and rearfoot (represented by areas A, B, and C, respectively). Based on Equation (1), subjects were classified into three groups. The first group comprised mainly those members diagnosed with high arches if his (or her) AI value was less than or equal to 0.21. Those members diagnosed with low arches comprised the second group when the AI value was greater than or equal to 0.26. The last group included those members diagnosed with normal arches if the AI value was between 0.21 and 0.26.

AI = B/(A + B + C)(1)

The AI values of these participants are shown in Table 1 and Table 2 with the following demographic information: Male: 25, Female: 1; Age: 22 ± 1 years; group I: height 175.5 ± 6.6 cm, body mass 72.3 ± 12.2 kg, BMI 23.4 ± 4.6; group II: height 172.1 ± 4.8 cm, body mass 69.5 ± 8.4 kg, BMI 23.4 ± 4.1; group III: height 173.6 ± 2.5 cm, body mass 68.3 ± 5.4 kg, BMI 22.6 ± 1.2; and group IV: height 172.1 ± 5.6 cm, body mass 73.3 ± 6.5 kg, BMI 24.7 ± 2.5. Significant differences were not found in the body mass indexes of the four groups of people.

### 2.2. Materials

#### 2.2.1. MP-5 Footprint Device

The ink footprint is a valid, simple, inexpensive, and noninvasive method. This concept is widely applied in clinical practice to not only study foot structures but also to explore flatfoot and diagnose pathologic conditions [25]. To better obtain information about the sizes of the three different zones (A, B, and C) in each subject’s feet, an MP-5 footprint device (Vers Technology Company/Taipei/Taiwan) was utilized to collect the subjects’ footprints. Sixty-five subjects were invited to receive the ink footprint test. The darker the footprint, the greater the pressure exerted. Then, we used AutoCAD software to detect the contours of the three zones.

#### 2.2.2. MP-1 Plantar Pressure Test Strip

To understand the distribution of the most stressful points for subjects with either flat feet or normal feet, 10 participants (5 of whom had two flat feet, and 5 of whom had two normal feet) were invited to walk 250 steps while wearing the MP-1 plantar pressure test strip (Vers Technology Company/Taipei/Taiwan). The plantar pressure test strip showed relatively high foot pressure, and it was demonstrated in the following three blocks, namely, the hallux, forefoot, and rearfoot. The difference between flat feet and normal feet was observed in the arch of the foot, which is located in the midfoot.

#### 2.2.3. Homemade Foot Pressure Sensing Insoles

**Figure 1:**
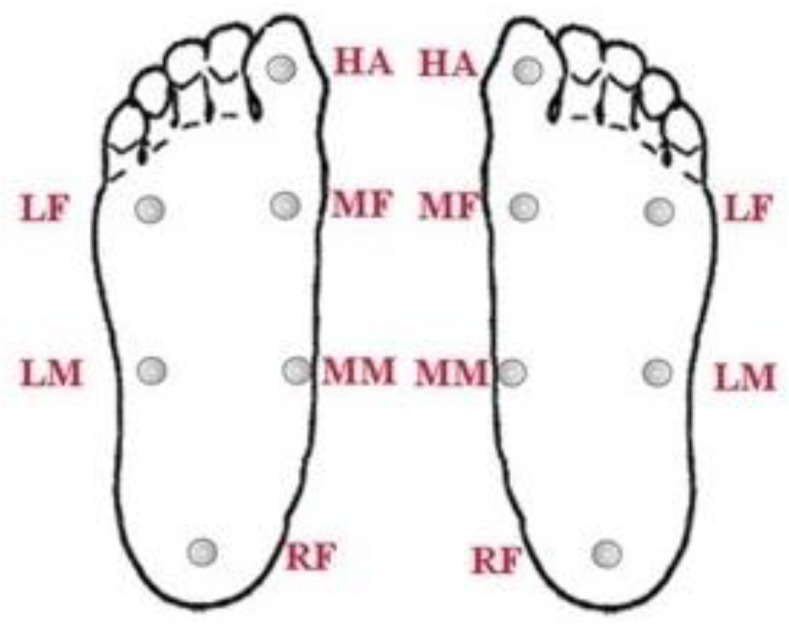
Blocks of the left and right plantar surfaces of the foot.

Based on the results of Section 2.2.1 and Section 2.2.2, we divided the plantar surface of flat and normal feet into 6 blocks: the hallux (HA), medial forefoot (MF), lateral forefoot (LF), medial midfoot (MM), lateral midfoot (LM), and rearfoot (RF). As shown in Figure 1, the two feet had a total of 12 blocks. Each block was designed with a piezoresistive sensor (Tekscan Company, South Boston, MA, USA). This was used to collect data on plantar pressure from the subjects participating in this study. Twenty-six subjects volunteered to participate in this homemade foot pressure sensing insoles study.

The research team employed a 3D printer to design three textured insoles with different particle heights (0, 3, and 6 mm) and artificial arch supports (7 cm long, 1.5 cm wide, and 1.5 cm high) for self-creation. Each textured insole was designed to include 18 textured granules of the same height and size. Based on the experimental results derived from the plantar pressure test strips, the maximum pressure of the participants was observed in the forefoot area and the second-largest pressure in the area of the HA and RF. According to the granule placement, the pressure on the RF caused instability and risk of a fall. Additionally, the forefoot occupied the largest area of contact when the foot was in contact with the ground during the stance phase. Therefore, we only placed textured granules under the forefoot. The artificial arch support was placed on the MM. All of the experiments were conducted when the subjects were requested to step barefoot.

### 2.3. Methods

**Figure 2:**
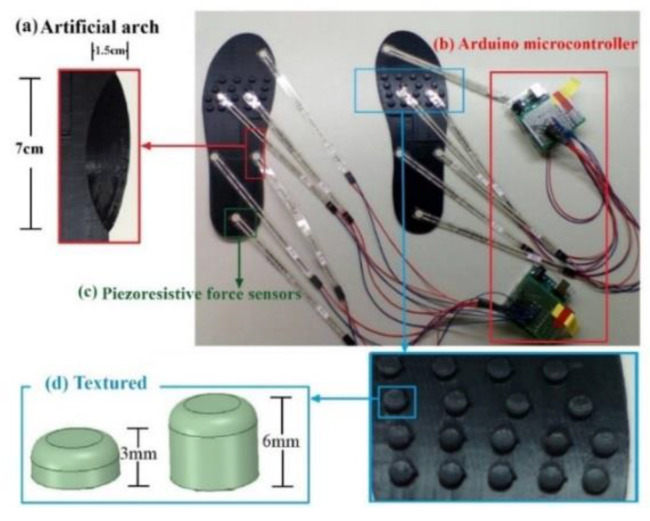
Homemade foot pressure sensing insoles.

The subjects were invited to wear the foot pressure insoles with three different heights and the arched insoles (Figure 2). In each individual experiment, each participant was asked to wear the equipped insole for 60 s. SPSS version 26 statistical software was used for statistical analysis. The statistical analysis of variance (ANOVA) was employed for experimental data analysis of the flat feet and normal feet. To be specific, in this study, a *p*-value less than 0.05 indicated a significant difference between the data of the experimental group and those of the control group. In addition, the study used three additional measures: (1) mean pressure (MP) represents the average plantar pressure value that fluctuated up and down during the collection of data; (2) peak pressure (PP) is the maximum instantaneous plantar pressure value; and (3) the value of the standard deviation (STD) represents the fluctuation of the pressure obtained by each pressure point during the collection of the data in relation to the mean pressure (MP).

## 3. Results

### 3.1. Difference in Plantar Pressure between Subjects with Fallen Arches in Both Feet and Those Only with One Flat Foot

**Figure 3:**
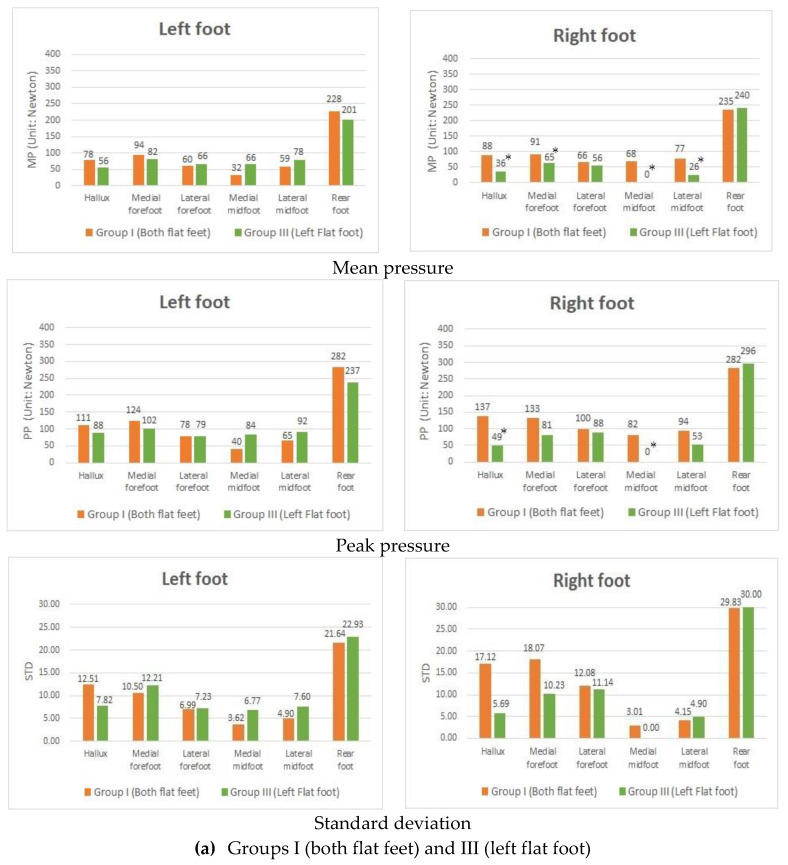

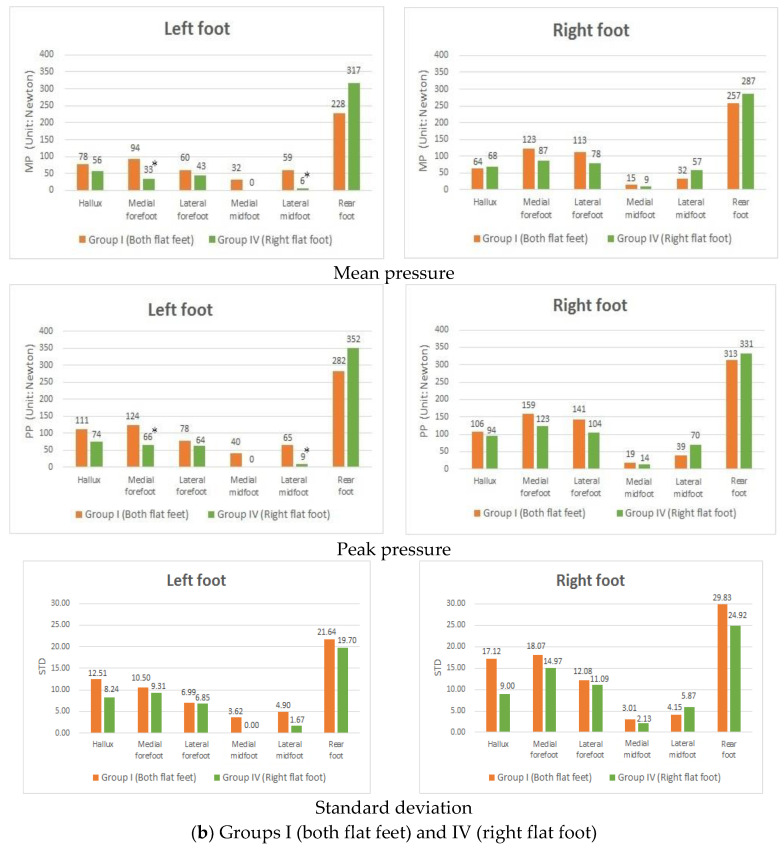
Mean pressure (MP), peak pressure (PP), and standard deviation (STD) values in participants with two flat feet (Group I), with a left flat foot (Group III), and with a right flat foot (Group IV) with the 0 mm insole. * *p*-value < 0.05, indicates that a significant difference exists.

The purpose of this study was to examine the difference in foot pressure between subjects with fallen arches in both feet and those with only one flat foot. Among the participants, some were diagnosed with fallen arches in both feet and so referred to as group I. In contrast, others diagnosed with only one flat foot were put into groups III and IV. The experiments in this study included two major types. Concerning the first experiment, participants in groups I and III were invited to wear the homemade pressure-sensing foot insoles with no textured heights and arches. We noted that the difference between these two groups was that, for people in group I, the right foot was flat, while, in contrast, those people in group III had a normal right foot. It is also worth noting that for the subjects in both groups, the left foot was flat. The results of groups I and III (Figure 3a) showed that the MP and PP in people diagnosed with fallen arches in both feet were relatively higher than those people with a single left flat foot. In particular, there are significant differences in the HA, MF, MM, and LM in the right foot. In the second experiment, participants in groups I and IV (Figure 3b) had significant differences in MP and PP, particularly in the areas of the MF and LM in the left foot. The conclusion is consistent with our result in the previous section that people in the group with flat feet tended to place their weight on the front of their feet, causing them to lean forward while standing. Noticeably, participants diagnosed as having fallen arches in both feet tended to have this more often than those with one flat foot.

Another major difference was that the STD (Figure 3a) generated from people with fallen arches in both feet at the areas of the HA, MF, LF, and MM in the right foot were higher than those people diagnosed with a single flat foot, which indicates that the defect of the arch caused poor body stability when it comes to standing and stretching exercises. In the second experiment, the STD showed in groups I and IV (Figure 3b). The result was similar to that obtained from the first experiment. Group III and IV had one normal foot, so their MM showed almost zero pressure in their normal foot. This finding implies that regardless of whether people had two flat feet or only one flat foot, there was no significant difference in their foot pressure on the side with the flat foot. Based on the abovementioned findings, the following experiments only consider one flat foot.

### 3.2. Difference in Plantar Pressure between Participants with Both Flat Feet and with Both Normal Feet

**Figure 4:**
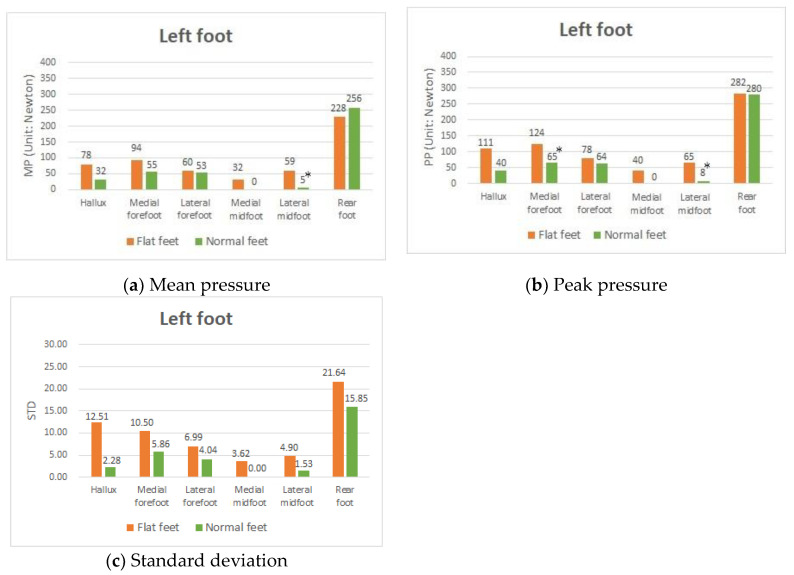
MP, PP, and STD values in participants with two flat feet and two normal feet with the 0 mm insole in the left foot. * *p*-value < 0.05, indicates that a significant difference exists.

The present study sought to investigate whether there are differences demonstrated in the plantar pressure between the group members with flat feet and those with normal feet. Nine participants had fallen arches in both feet (group I), and nine had two normal feet (group II). Because the same experiments were conducted with the left and right foot, we chose the left foot for data analyses. In this experiment, these groups of people were requested to wear the 0 mm insole with no arch support attached, and the results derived from this experiment were then compared. A general finding was that, for people with fallen arches in both feet and those with normal feet, the highest MP and PP were found in the RF of the left foot, indicating that people tended to put weight on the RF (Figure 4a,b). An obvious difference, as expected, was the plantar pressure difference in the midfoot area (MM and LM).

People in the group with flat feet showed high MP and PP, while, in contrast, people in the group with normal feet showed almost zero pressure. This was because the former group had the arch deformity problem, whereas the latter had an arch to support their body weight. An interesting result was that the plantar pressures in the participants with flat feet were comparatively higher than those in the participants with normal feet in the HA and MF.

Notably, both the MP and PP in the participants with flat feet in these two areas were almost double those observed in the participants with normal feet. In particular, the MF was significantly different in the PP. The results implied that those group members diagnosed with flat feet tended to put their weight on the front of their feet, causing them to lean forward while standing due to the defect of the arch. In the long run, this might cause substantial damage to the HA and MF in flat feet, which in turn could possibly cause these people to suffer from hallux valgus and plantar fasciitis.

The results showed that the STD value generated from the group members with flat feet was higher than that generated from the group members with normal feet (Figure 4c). The STD value of the group with flat feet in the HA was approximately 5.5 times the STD value of the group with normal feet (flat feet: ±12.51; normal feet: ±2.28). In addition, the STD value of the group with flat feet in the MF was 79% higher than the STD value of the group with normal feet (flat feet: ±10.50; normal feet: ±5.86) in the left foot. Thus, the defect of the arch not only caused an abnormal distribution of foot pressure but also resulted in poor body stability in standing and stretching positions or exercise.

### 3.3. Effects of Using the Textured Insoles with Different Heights on Plantar Pressure in Both Flat Feet and Both Normal Feet

**Figure 5:**
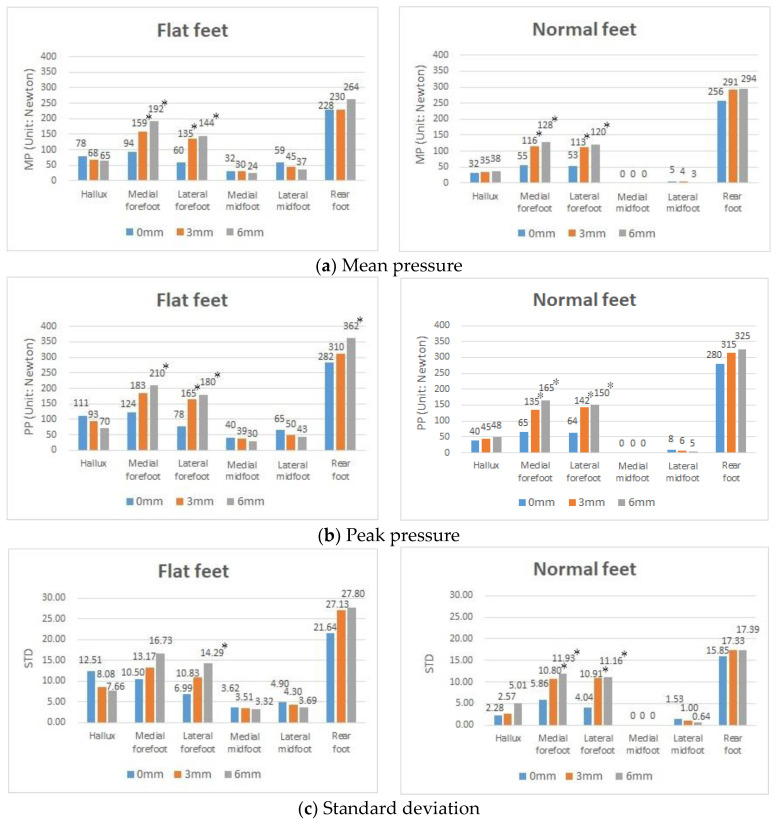
Figure showing the comparison of foot pressure distribution with textured insoles. * *p*-value < 0.05, indicates that a significant difference exists.

In this experiment, participants were required to wear insoles of three different heights, i.e., 0, 3, and 6 mm, independently, and were given no arch support. For people with normal feet, the experimental results (Figure 5a,b) showed that the MP and PP at each contact point increased when the heights of the textured insoles increased, indicating the textured insole directly increased the effectiveness (or sensitivity) of detecting plantar pressure. Similarly, for the group diagnosed with flat feet, the MP and PP were observed to rise at the contact points of the textured insoles (i.e., the forefoot areas) as well as the RF area. However, in contrast, the MP and PP in the areas of the HA and midfoot (MM and LM) decreased when the heights of the textured insoles were increased.

This finding suggests that using the textured insoles designed with an appropriate height would further help reduce high plantar pressure problems under the HA and midfoot (MM and LM) for those who have flat feet. Another interesting result (Figure 5c) was that the STD of the participants with flat feet decreased in the HA as the height of the textured insoles increased, suggesting that textured insoles of an appropriate height not only reduces MP and PP but also decreases the dynamic instability of the HA area.

### 3.4. Effects of Using the Foot Arched Insoles on Plantar Pressure in People with Both Flat Feet

**Figure 6:**
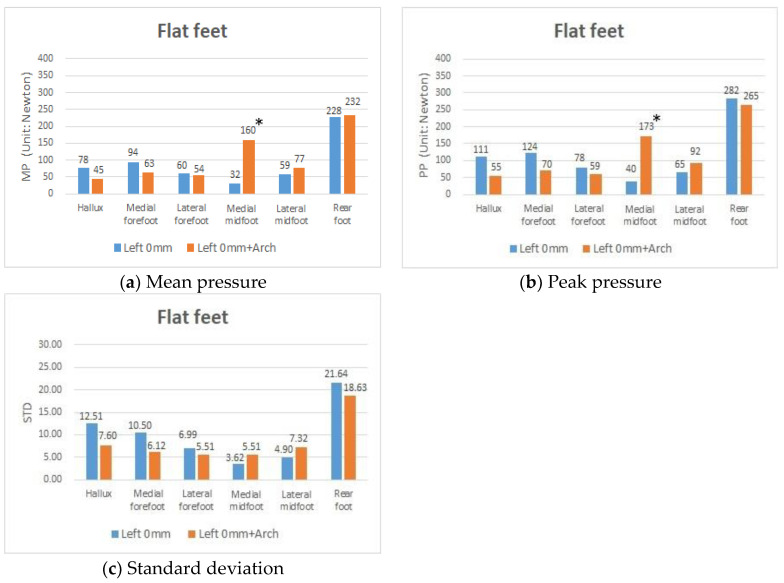
MP, PP, and STD values in participants with both flat feet, with the 0 mm insole in the left foot and with the 0 mm insole + arch insole in the left foot. * *p*-value < 0.05, indicates that a significant difference exists.

Using an artificial arch (height of 0 mm with and without arch support), the results showed that MP in people with fallen arches in both feet (Figure 6a) decreased from 78 to 45 in the HA, from 94 to 63 in the MF, and from 60 to 54 in the LF. In terms of PP (Figure 6b), there was a decrease from 111 to 55 (50% reduction) in the HA, from 124 to 70 (43% reduction) in the MF, and from 282 to 265 (6% reduction) in the RF. In contrast, there were significant increases in the MP from 32 to 160 and in the PP from 40 to 173 in the MM due to using an artificial arch. The results showed that the STD value generated from those people with the collapse of arches in both feet decreased in the HA, MF, LF, and RF. All of these findings supported the view that when an artificial arch was used, the chance of substantial plantar pressure damage might be greatly relieved and physical stability in flat feet effectively improved.

**Figure 7:**
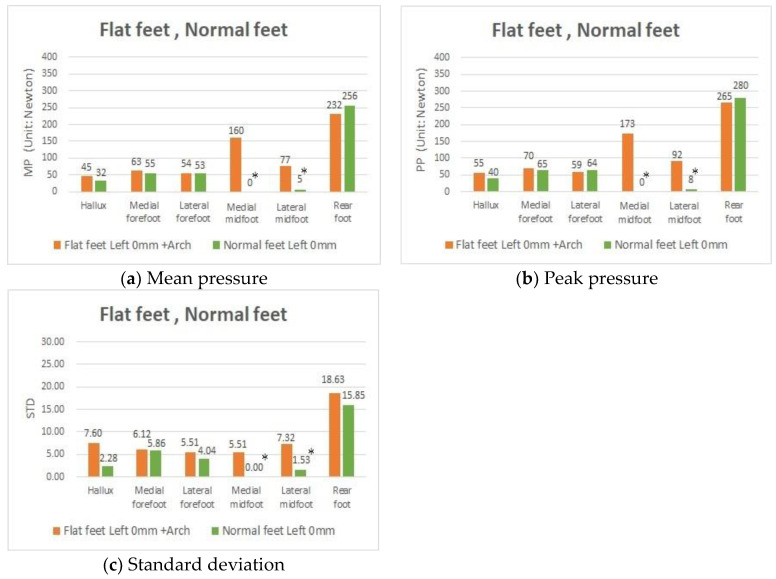
MP, PP, and STD values in participants with both flat feet and both normal feet with the 0 mm insole and the 0 mm insole + arch insole in the left foot.

A further study was needed to investigate whether there are significant differences in the values of MP, PP, and STD between people provided with arch support because of fallen arches and people with normal feet. The results (Figure 7) showed that these two groups of people had almost the same MP, PP, and STD in all areas, except for the two points of the MM and LM. The differences in the latter areas were mainly because of the pressure from the arch insoles, indicating that the flat feet could be restored to normal pressure distribution and body stability through arch insole corrections.

## 4. Discussion

This study aimed to explore whether there is a difference in plantar pressure between subjects with fallen arches in both feet and those with only one flat foot. According to our survey, we believe that people in the group with flat feet tended to place their weight on the front of their feet, causing them to lean forward while standing. The phenomenon was more notable for participants who had two flat feet than in participants with one flat foot. The results showed that the MP, PP, and STD in people diagnosed with fallen arches in both feet were relatively higher than those people with a single flat foot.

We studied the differences demonstrated in plantar pressure between people with flat feet and those with normal feet. The results showed that, when compared with people with normal feet, those with flat feet had a comparatively higher MP, PP, and STD in the HA and forefoot (MF and LF). It was found that people diagnosed with flat feet tended to lean forward while standing or walking. The RF represents the block where people with flat feet and normal feet are subjected to maximum plantar pressure when standing, and it is also the most important block for observing body balance. The MP of people with flat feet was comparatively lower than that of people with normal feet in the RF. The above result is consistent with that of Jin et al. [3], who found the peak plantar pressure of the flat feet group was lower than in the normal feet group in the heel region but higher in the big toe area. However, by contrast, the PP of the former group was higher than that of the latter group. In particular, the plantar pressure distribution in subjects with flat feet changed continuously during the test process. This helped to reveal subjects’ poor body stability when they were standing and stretching.

The results of this study demonstrated that the use of textured insoles had a beneficial influence on people diagnosed with flat feet, and the problem in relation to this was minimized to a certain extent. This phenomenon could be moderately improved by using textured insoles. However, the increase in insole height may not necessarily help this problem. We noted that our above result was in accordance with the finding of Chen et al. [16] that using textured insoles helped increase pressure in the midfoot area but decreased pressure in the toe area. When the arched insoles were placed on the MM contact areas of the subjects with flat feet, their plantar pressure distributions were quite similar to those of the subjects with normal feet. This indicates that participants with flat feet could experience a normal pressure distribution using arched insoles, thus improving their body stability through arched insole corrections. Arched insoles can be a successful treatment to reduce the symptoms of flat feet. The conclusion is similar to that in Chen et al. [22] and Nakajima et al. [23]. Table 3 includes a summary of the comparison of the proposed method with other literature.

## 5. Conclusions

Footwear manufacturers have faced the challenge of fitting shoes accurately for various foot types. With this in mind, the results of the present study could not only provide another source of information to footwear manufacturers but also increase the possibility of the product development of customized insoles. In addition, a commercially available plantar pressure analysis system can cost between USD 10,000 and 20,000, making it unaffordable for patients. In this study, we designed a low-cost homemade foot pressure sensing device (only a few hundred dollars) equipped with textured insoles of different heights and artificial arches to explore the effects of artificial texture insoles and foot arches on improving arch collapse in flat feet. Although the homemade insole sensing device designed by this institute is not a high-precision instrument, it helps to provide a simple and relatively low-cost tool for patients in need, and this concept could possibly allow the majority of scholars to invest time and energy in this field of research. In this case, this could also enhance public awareness in relation to affordable self-testing tools. It is hoped that when attention is drawn to the research advances in this field, possible increased cooperation with hospitals to assist in clinical diagnosis through this instrument would be encouraged. In addition, in the future it is also hoped that sufficient data using this technique will be collected to allow artificial intelligence systems to be incorporated into this field of study to further capture the specific biological characteristics of individuals. The aim would be to generate “sensing and timely” signal notification, for the system as presented is believed by the authors to reduce possible foot damage for patients.

## Figures and Tables

**Table 1:** The arch index (AI) of subjects in group I and group II.

	Subjects	Classification	AI of the Left Foot	AI of the Right Foot
Group I	A	Both Flat Feet	0.353	0.293
	B		0.291	0.308
	C		0.354	0.261
	D		0.374	0.267
	E		0.261	0.275
	F		0.356	0.336
	G		0.276	0.306
	H		0.293	0.266
	I		0.265	0.263
Group II	J	Both Normal Feet	0.229	0.228
	K		0.259	0.251
	L		0.254	0.254
	M		0.250	0.256
	N		0.244	0.238
	O		0.242	0.255
	P		0.216	0.259
	Q		0.238	0.236
	R		0.257	0.259

**Table 2:** The AI of subjects in group III and group IV.

	Subjects	Classification	AI of the Left Foot	AI of the Right Foot
Group III	S	Left Flat Foot	0.353	0.256
	T		0.303	0.247
	U		0.293	0.251
Group IV	V	Right Flat Foot	0.235	0.263
	W		0.249	0.300
	X		0.241	0.289
	Y		0.217	0.267
	Z		0.239	0.265

**Table 3:** Comparison of research findings and literature review.

Research Findings	Author	Data Collection	Literature Review
The results showed that, when compared with people with normal feet, those with flat feet had a comparatively higher MP, PP, and STD in the HA and forefoot (MF and LF). The MP of people with flat feet was comparatively lower than that of people with normal feet in the RF.	Jin, T. H.; Hyun, M.K.; Jae, M. J.; Yeun, J.K.; Jung, H.L. (2011) [3]	Matscan system	In the heel region, the peak plantar pressure of the flat feet group was lower than in the normal feet group and the difference was statistically significant (*p* < 0.05). In the big toe area and the small toe area, peak plantar pressure of the flat feet group was higher than in the normal feet group, but without significant difference.
The results showed the MP and PP in the areas of the HA (hallux) and midfoot (MM and LM) decreased when the heights of the textured insoles were on the increase.	Chen, H.; Nigg, B. M.; Hulliger, M.; Koning, J. D. (1995) [16]	Flexible pressure measuring insole	The pressure increased in the midfoot area and decreased in the toe area with increasing sensory inputs.
The results showed the use of an artificial arch effectively improved the excessive peak in pressure, poor body stability, and alleviate the problem of plantar collapse for patients with flat feet, especially in the inner part of their hallux and forefoot.	Chen, Y. C.; Lou, S.Z.; Huang, C.Y.; Su, F.C. (2010) [22]	Kistler force plates	The results suggested that the foot insoles and shoes developed in this study might benefit the ankle joint in patients with flat feet.
The results showed the use of an artificial arch effectively improved the excessive peak in pressure, poor body stability, and alleviate the problem of plantar collapse for patients with flat feet, especially in the inner part of their hallux and forefoot.	Nakajima, K.; Kakihana, W.; Nakagawa, T.; Mitomi, H.; Hikita, A.; Suzuki, R.; Akai, M.; Iwaya, T.; Nakamura, K.; Fukui N. (2009) [23]	Kistler force plates	Addition of an arch support to the laterally wedged insole reduced knee adduction moment more efficiently, possibly through the elimination of potential negative effects of the laterally wedged insole.

## Abbreviations

The following abbreviations are used in this manuscript:

AI	arch index
HA	hallux
LF	lateral forefoot
LM	lateral midfoot
MF	medial forefoot
MM	medial midfoot
MP	mean pressure
PP	peak pressure
RF	rearfoot
STD	standard deviation
